# Effective Social Support to Enable Older Adults Living Alone in Japan to Continue Living at Home

**DOI:** 10.3390/ijerph22071084

**Published:** 2025-07-07

**Authors:** Miwako Naoe, Yasuhiro Kawahara

**Affiliations:** Department of Human Life and Health Sciences, Graduate School of Arts and Sciences, The Open University of Japan, Chiba 261-8586, Japan

**Keywords:** older people, living alone, living at home, nursing care level, care service

## Abstract

Japan has the world’s fastest-aging population. The number of older adults living alone has increased rapidly; however, the number of people waiting for nursing care facilities is high, especially in urban areas, and home care is unavoidable. Few studies have focused on older adults living alone who require nursing care, and almost no reports have examined the factors influencing the continuation or discontinuation of living alone. Furthermore, no reports were found that classified nursing care levels for the survey. This study’s purpose was to clarify what kind of long-term care for older adults living alone in urban areas is most effective in enabling them to continue living at home. A total of 122 older adults in need of long-term care in Osaka City were divided into two groups; one group was those who continued to live alone in December 2022, the other was those who had to discontinue doing so after January 2020. A questionnaire was distributed to the care managers responsible for older adults living alone who required nursing care. The participants’ basic attributes, long-term care services usage, and the characteristics of instrumental activities of daily living (IADL) support were compared according to care level using Fisher’s exact test. The relevant effective factors for continuing to live alone were extracted using a multivariate logistic regression analysis. The results showed differences in the characteristics of both groups at both care level categories used in the study, Support Care Level 1–Nursing Care Level 2 and Nursing Care Level 3–5. Among the support items, indoor temperature control was suggested as a factor that influences continued living alone.

## 1. Introduction

Ten percent of the world’s population is aged 65 years and older, and this proportion is increasing. Regarding the percentage of national populations composed of older adults, Japan has the highest (29.1%) worldwide, followed by Italy (24.5%), Finland (23.6%), Germany and France (>20%), South Korea, (18.4%), and China (14.3%) [1].

Hence, Japan has the highest proportion of older adults worldwide. Furthermore, the number of older people aged 65 years and older living alone is increasing amid rapid aging and a shift to nuclear families. This proportion was 15.0% for men and 22.1% for women and is estimated to increase to 20.8% for men and 24.5% for women by 2040, with urban areas showing an accelerating trend [2].

### 1.1. Global Trends in Home Care for Older Adults Living Alone

The number of older adults living alone is also increasing in Europe, and home care is becoming increasingly more common than institutional care [3]. In Italy, the majority of older adults prefer to stay at home with a caregiver and consider nursing homes as a last resort [4]. Older adults also reported a lack of support from family members and low public-service provision levels. Furthermore, home-based services require strengthening [5]. In Finland, assisted-living homes are popular [6], and medical and social care for older adults is increasingly being provided at home [7].

Studies conducted in South Korea reported a lack of services that take into consideration the living environment and gender differences among older adults living alone [8]. An increase in the number of older adults living alone led to a rapid increase in home care in China. However, the utilization rate of care services and equipment was surprisingly low. In addition, social resources were unused, which suggested the need to reform care services [9]. In general, with the number of older people living alone increasing in aging countries, the need for in-home care will rise.

In Japan, a country with a rapidly aging population, a national policy was enacted in 2014 [10] to promote a regional comprehensive care system. Environmental improvements and measures for living at home were also implemented. There were approximately 4 million in-home service recipients and 950,000 institutional recipients in 2022 [11], and approximately 300,000 people were waiting for admission to institutions nationwide [12]. Notably, the more urban the area, the longer the waiting list. Approximately 70% of older adults living alone preferred home care when the level of care required was low. However, they preferred nursing homes once this level increased [13].

Older people who require nursing care and live alone need to continue living at home for as long as possible as finding an available place in a residential institution is difficult, even after they are unable to continue home care. The difficulties faced by older adults living alone and requiring care gradually become apparent in physical tasks such as shopping and meal preparation as well as in managing finances and medication. Since social resources and administrative budgets are limited, understanding what care services and daily living supports are effective at each stage of long-term care is necessary.

### 1.2. Home Care Situation of Older Adults Living Alone in Japan

This study focused on the relationship between continued living alone and the type of home care supports received by elderly persons in need of nursing care in Japan. Previous studies on the current situation of older adults who live alone and the challenges they face have often focused on social isolation [14]. Many have examined psychological aspects, such as depression, happiness, and cognitive function [15]. However, fewer studies have focused on the home life of older adults who live alone and require nursing care.

In Japan, the Ministry of Health, Labour and Welfare (MHLW) defines the levels of support and care required that apply to persons in need of nursing care in order to provide them with specific assistance. There are seven ranks of care levels, and the physical and mental state associated with each is described below.

- Support Care Level 1: Mostly independent in daily living and go out on their own although they have difficulty standing up smoothly by themselves.
- Support Care Level 2: Difficulty in getting up, standing on one leg, and shopping, but their condition may be alleviated by rehabilitation and other means.
- Nursing Care Level 1: Difficulty in getting up alone. Regularly forgetting directions or locations. Make frequent mistakes in tasks such as shopping, clerical work, and money management, which they were able to do before.
- Nursing Care Level 2: Need assistance with walking, washing, nail trimming, taking medication, managing money, and simple cooking.
- Nursing Care Level 3: Need assistance with urination, defecation, oral hygiene, putting on/taking off upper clothes, putting on/taking off pants, etc.
- Nursing Care Level 4: Need assistance turning over in bed, standing on both legs, transferring to a wheelchair, moving around, washing face, and washing hair.
- Nursing Care Level 5: Spend all day in bed and require assistance with toileting, eating, and changing clothes.

Otaga [16] demonstrated that even if a person without dementia lived alone and could not expect family care, they could live alone until they reach Nursing Care Level 2, based on a system that provides care, such as housework assistance and good management. However, many older people had stopped living alone by the time they reached Nursing Care Level 1. Horiguchi et al. [17] reported that 50% of older adults in the non-dementia group who discontinued living alone had care levels that ranged from Support Care Level 1 to Nursing Care Level 1. They stated that further identification of the factors that influenced the continuation of living alone was necessary along with a comprehensive examination of how support should be provided. Other studies suggested the need to utilize various care services [18,19,20]. However, no study has examined the extent to which the services are utilized by older adults who live alone and their different requirements according to their level of care.

In addition, older people with dementia experience functional decline owing to hospitalization or temporary physical changes. Returning to a familiar home environment and receiving appropriate support, including the use of assistive products, could lead to appropriate maintenance and recovery of function [21].

### 1.3. Purpose

The waiting list for nursing care facilities is longer in urban areas, and older people requiring nursing care who live alone are forced to live at home. Hence, enabling them to continue living at home is necessary. In addition, as people age, their ability to adapt to changes in their environment declines and confusion is more likely to occur owing to cognitive decline. Therefore, maintaining their ability to continue living in a familiar place is necessary to support peace of mind among older adults who live alone and require care. Clarifying the factors that make it difficult to live at home and those necessary to continue living alone could help keep older adults living at home for longer. Therefore, we need to examine which social resources they use and how often. The ways to improve their environment and further support for older adults should also be considered. This study aimed to investigate the use of nursing care services and daily living support for older adults who required nursing care and lived alone in urban areas, with a focus on the level of nursing care required. Furthermore, we also explored the factors that enabled them to continue living alone based on the differences in the characteristics between those who continued to live alone and those who had to discontinue doing so.

## 2. Materials and Methods

### 2.1. Participants

In this study, a mail survey was conducted using an anonymous, self-administered questionnaire sent to care managers who had experience of being responsible for elderly persons requiring nursing care who lived alone. First, a research cooperation request form was sent to managers of in-home care support facilities in Osaka City, and a research cooperation request form and a self-administered, anonymous questionnaire were distributed to those care managers who agreed to cooperate after an explanation from those managers who responded to the request for cooperation. If they consented to the survey, the care managers were asked to return the survey form directly to the researcher using the self-addressed envelope. The care managers explained the survey to the older adults in their charge and detailed the data concerning the attributes and care services of those elderly who gave their consent. The elderly included in the survey comprised those who were still living alone as of the end of December 2022 (“continuous” group) and those who had stopped living alone after January 2020 and were not living alone as of the end of December 2022 (“interrupted” group). For the older adults who had stopped living alone, the care managers answered by recalling their attributes and the care services provided immediately prior to the interruption. Interrupted cases were limited to those within three years to reduce recall bias. The study period was from January 2023 to February 2023, and 19 care managers cooperated in the survey.

### 2.2. Survey Items

Based on previous research [17,18], the questionnaire comprised questions regarding the participants’ basic characteristics, usage of long-term care insurance (LTCI) and non-insurance care services, contents of instrumental activities of daily living (IADL) support, and characteristics of the care manager in charge. The questionnaire was based on the Japanese version of Lawton and Brody’s IADL scale [22,23] and the Ministry of Health, Labour and Welfare’s survey data [24].

#### 2.2.1. Participants’ Characteristics

In total, seven items enquired whether or not they continued to live alone, their age and gender, nursing care level, degree of independent daily living for older adults with disabilities (eight ranks), degree of independent daily living for older adults with dementia (seven ranks), presence of medical treatment, and care supporters (family members or relatives living separately, community residents, welfare committee members, friends, care helpers, others, or none).

The ranks of degree of independent daily living for older adults with disabilities are as follows:

- Rank J1: They have a disability of some kind but are mostly independent in daily life and go out by using public transportation, etc., on their own.
- Rank J2: They have a disability of some kind but are mostly independent in daily living and can go out on their own in the neighborhood.
- Rank A1: They are generally independent in indoor living but do not go out without assistance. They require assistance when going out and live away from their bed most of the time during the day.
- Rank A2: They are generally independent in indoor living but do not go out without assistance. They go out infrequently and sleep and wake up during the day.
- Rank B1: They need some kind of assistance to live indoors. They mainly stay in bed during the day but maintain a sitting posture. They transfer to a wheelchair by themselves and eat and use the toilet away from the bed.
- Rank B2: They need some kind of assistance to live indoors. They mainly stay in bed during the day but maintain a sitting posture. They can transfer to a wheelchair with assistance.
- Rank C1: They spend all day in bed and require assistance with toileting, eating, and changing clothes. They turn over in bed on their own.
- Rank C2: They spend all day in bed and require assistance with toileting, eating, and changing clothes. They cannot turn over in bed without assistance.

The ranks of degree of independent daily living for older adults with dementia are as follows:

- Rank I: They have some form of dementia but are largely independent in daily life both in the home and socially.
- Rank IIa: They can be independent outside the home, even if some symptoms/behaviors or communication difficulties that interfere with daily life are observed, if someone is minding them.
- Rank IIb: Even if some symptoms/behaviors or communication difficulties that interfere with daily life are observed, they can be independent if someone is minding them.
- Rank IIIa: They require nursing care, mainly during the day, due to symptoms/behaviors and communication difficulties that interfere with daily life.
- Rank IIIb: They require nursing care, mainly during the night, due to symptoms/behaviors and communication difficulties that interfere with daily life.
- Rank IV: Symptoms and behaviors that interfere with daily life and communication difficulties are frequently observed, requiring constant care.
- Rank M: Significant psychiatric or peripheral symptoms or serious physical illness are observed, requiring specialized medical care.

#### 2.2.2. Use of LTCI and Non-Insurance Care Services

The participants were asked to report the frequency of use of 11 items (home-visit care, home-visit bathing service, home-visit nursing care, management guidance for in-home care, commuting for care, commuting for rehabilitation services, home-visit rehabilitation, short-term stay at a care facility, meal delivery service, eating out, and use of take-out lunch boxes and prepared foods) and their use of three services (temporary provision of assistive products, house modifications, and emergency alert equipment). For nursing care prevention support (exercise, hobbies, social events, and others), the respondents were asked to suggest the content. These 15 items were used to assess the use of LTCI and non-insurance care services.

#### 2.2.3. IADL Support

The participants were asked regarding the presence or absence of the following 11 items: money management, temperature control, meal preparation, cleaning, laundry, medication management, garbage disposal, sanitation, oral care, talking to others, and frequency of outings.

#### 2.2.4. Characteristics of Care Managers

The care managers’ age, gender, and years of experience were obtained.

### 2.3. Analysis

The survey answers were converted into numerical data, and the missing values were indicated as NA. To compare the “continuous” and “interrupted” groups, differences between their characteristics, use of LTCI and non-insurance services, and content of IADL support were analyzed using Fisher’s exact tests. The difference in utilization rates between the continuous and interrupted groups in care support items related to continued living alone was estimated to be 30%. Assuming a two-sided alpha of 0.05 and a statistical power of 80%, and considering that the number of people in the interrupted group was approximately one-third of the continuous group during the survey period, the required sample sizes for the continuous and interrupted groups were calculated to be 81 and 27, respectively. Continuation/interruption of living alone was set as the dependent variable, and characteristics, frequency of use of LTCI and non-insurance services, and presence/absence of IADL support were set as independent variables.

In addition, to compare the differences according to nursing care level, Fisher’s exact test was used to analyze the differences within each of the “continuous” and “interrupted” groups in terms of their characteristics, use of LTCI and non-insurance care services, and IADL support on the basis of their care level, divided between those of Nursing Care Level 2 or lower (Support Care Level 1–Nursing Care Level 2), and those of Nursing Care Level 3 and above (Nursing Care Level 3–5). Explanatory variables that considered factors regarding continuing to live alone were selected.

A multivariate logistic regression analysis was conducted to identify the factors that influenced continuing to live alone. Significance was set as *p*-values of <5%. EZR (Ver.1.61, Jichi Medical University, Tochigi, Japan), a statistical software that extended the capabilities of R and R Commander, was used for all statistical analyses [25].

### 2.4. Ethical Considerations

This study was approved by the Research Ethics Committee of the Open University of Japan (Approval No. 2022-49-54, approved on 15 December 2022). The research cooperation request form presented to all care managers clearly explained the study purpose and methods, data anonymization, processing of the results, and that no one would be disadvantaged if they did not participate.

## 3. Results

### 3.1. Characteristics of Care Managers and Older Adults in Their Charge

Nineteen care managers cooperated in the study. The relationship between the care managers’ ages, genders, and years of experience and the older adults surveyed by the care managers is shown in Table 1. There were no significant differences in care manager characteristics between the continuous and interrupted groups; 60% had five or more years of experience, and 80% were women aged 40 years or older. In this study, information on these older adults was used in the analysis. Of the 125 sets of data on older adults obtained in the survey, three were excluded as the response form did not indicate if they belonged to the continuous or interrupted group. Of the 122 valid responses, 90 belonged to the continuous group and 32 to the interrupted group. It should be noted that the totals in the tables do not always add up to 90 and/or 32 due to individual missing data items.

### 3.2. Differences in Participants’ Characteristics Between the Continuous and Interrupted Groups

Table 2 presents a comparison of the characteristics of older adults living alone, divided into two groups: those who have lived alone continuously and those who have lived alone interrupted. Significant differences were observed in nursing care level, degree of independent daily living for older adults with dementia, presence or absence of medical treatment, and care supporters. The continuous group was characterized by various care supporters, mainly those at Nursing Care Level 2. In the interrupted group, 40% received medical treatment, mainly those at Nursing Care Level 3.

### 3.3. Differences Between the Continuous and Interrupted Groups in Use of Long-Term Care Services

Table 3 presents the utilization of LTCI and non-insurance care services. Significant differences were observed in management guidance for in-home care, emergency alert equipment, temporary provision of assistive products, and meal delivery services. Furthermore, the percentage of use was higher in the interrupted group than in the continuous group. In terms of assistive product provision, the utilization rate was high in both the continuous group (62.2%) and the interrupted group (84.4%), whereas in meal delivery services, the utilization rate was low in both the continuous group (18.9%) and the interrupted group (34.4%). The characteristics of care service use were more varied and frequent in the interrupted group.

### 3.4. Differences in Support Between the Continuous and Interrupted Groups Regarding IADL

As shown in Table 4, in IADL support, significant differences were observed between the groups in 6 of the 11 items. Furthermore, the percentage of support in the interrupted group was higher for 10 items, with support for temperature control, oral care, meal preparation, garbage disposal, medication management, and laundry showing significant differences. The items of talking to others, assistance with outings, and cleaning had high support rates in both groups, with no significant differences.

Regarding the characteristics of support in activities of daily living, in the continuous group more people required communicative support, such as talking and going out. In the interrupted group more people required hygiene support, such as cleaning, garbage disposal, laundry, and oral care, and 90% required temperature control support.

### 3.5. Differences Between the Support Care Level 1–Nursing Care Level 2 and Nursing Care Level 3–5 Categories

Table 5 shows the use of services by the continuous and interrupted groups for items that were significantly different in at least one of these two care level categories, Support Care Level 1–Nursing Care Level 2 and Nursing Care Level 3–5.

In the Support Care Level 1–Nursing Care Level 2 category, significant differences were observed in temperature control, oral care, medical treatment, home-visit nursing care, management guidance for in-home care, and medication management. In the Nursing Care Level 3–5 category, significant differences were observed in temperature control and management guidance for in-home care. The use of other medical services and daily life support had almost the same characteristics in the continuous and interrupted groups.

### 3.6. Factors That Influenced Continuous Living Alone

To understand the factors that influenced continuing to live alone in older people who required nursing care and lived alone, a logistic regression analysis was conducted with the objective variable set as to whether they continued to live alone. Furthermore, seven items with high significance, likely to be highly relevant and confounding factors, were selected as independent variables. Table 6 presents the results of the analysis and it can clearly be seen that temperature control significantly influenced the continuation of living alone. Regarding temperature control, the odds ratios (ORs) and confidence interval were 0.06 [0.01–0.24]. The probability of continuing to live alone decreased without temperature control, indicating that temperature control affected the ability to continue living alone. The *p*-value of the likelihood ratio test is small, indicating that the model is useful, and since the variance inflation factors (VIF) were all less than 2, no multicollinearity issues were found. AIC was 107.07.

## 4. Discussion

### 4.1. Factors Related to Continued Living Alone Regarding Characteristics

Regarding the distribution of the number of persons between the nursing care levels, the continuous group had the highest number of those at Nursing Care Level 2, while the interrupted group had the highest number of those at Nursing Care Level 3. This represented a significant difference between the two groups and was similar to the results reported by previous studies [16]. The percentage distribution of Osaka City’s certified care-dependent population by care level (Support Care Level 1 to Nursing Care Level 5) was as follows: 0.197, 0.140, 0.136, 0.168, 0.128, 0.134, and 0.097 [26]. The sample for this study consisted of older adults living alone who received support from care managers, and the proportions of those classified as Support Levels 1 and 2, as well as Care Levels 4 and 5, are lower than the overall proportions for Osaka City.

Regarding the timing of the interruption of living alone, Horiguchi et al. [17] reported that 50% of older adults in the non-dementia group who discontinued living alone ranged from Support Care Level 1 to Nursing Care Level 1. Conversely, in this study the equivalent rate was 12.5% and the rate for those from Support Care Level 1 to Nursing Care Level 2 was 40%, suggesting that the period during which they could continue to live alone was longer than that highlighted in the previous study.

Medical treatment was received by 20% and 40% of those in the continuous and interrupted groups, respectively, which suggests an increase in home medical services as the level of care required increased, which is similar to the results of Maruyama’s study [27]. However, since a significant difference in medical treatment was observed between the continuous and interrupted groups in the Nursing Care Level 2 or below category, we believe that further research on the content of medical care is warranted.

Regarding supporters, more than 80% of those in both groups were supported by family members or relatives who lived separately from them. However, the continuous group had various supporters, such as friends, local residents, welfare committee members, and helpers. This suggested that receiving support without relying on relatives may help them to continue living alone.

### 4.2. Factors Related to Continued Living Alone Regarding the Use of Long-Term Care Services

In relation to the use of long-term care services, a significant difference between the continuous and the interrupted group was observed in the usage frequency of management guidance for in-home care services, and each group, according to the level of care required, showed the same significant difference in that usage frequency. Although significant differences were observed in the use of emergency alert devices, the number of users was small (1.1% for the continuous group, 16.1% for the interrupted group), which suggests that awareness of risk management was low. Further studies are required on the installation of emergency alert devices.

Significant differences between the groups were also observed in the use made of the temporary provision of assistive products and meal delivery services. For the assistive products, both groups showed a high utilization rate (62.2% for the continuous group, 84.4% for the interrupted group), a trend similar to that observed in a previous study [28], and the necessary assistive products seem to be used effectively. Regarding meal delivery services, the interrupted group had a higher frequency of use (18.9% for the continuous group and 34.4% for the interrupted group), but the use of lunch boxes and prepared foods was higher for both groups, at 50%. The rate of eating out was 20% in the continuation group and 10% in the interrupted group. The results showed that the percentage of people aged 60 and over who used lunch boxes and prepared foods was the highest, followed by eating out, while meal delivery services accounted for less than 10%, which was similar to the report of the Ministry of Health, Labour and Welfare’s “The National Health and Nutrition Survey in Japan” in 2019 [24]. The use of self-selectable lunch boxes, prepared foods, and “meal preparation” in daily living assistance was higher, which may indicate older adults’ commitment to food.

### 4.3. Factors Related to Continued Living Alone Regarding IADL Support

Temperature control and oral care support showed particularly large differences between the two groups. In particular, temperature control services showed a higher utilization rate (28.9% for the continuous group, 87.1% for the interrupted group) in the interrupted group and the logistic regression analysis suggests that support for temperature control is a factor influencing the continuation of living alone. Comparing the continuous and interrupted groups separately by nursing care levels, there was a significant difference between the groups for both Nursing Care Level 2 or below and Nursing Care Level 3 or above. This suggests that it is difficult to control the temperature throughout the day when living alone, that temperature control care is required even at the earliest levels of support and nursing care, and that temperature control is a related factor in continuing to live alone. Inadequate air-conditioning management in mid-summer can raise concerns regarding the possibility of people falling ill due to heatstroke, and older adults may consider giving up living alone when faced with illness. Over 80% of all heatstroke-related deaths in 2020 were among older adults. Causes at home were attributed to the short duration of air-conditioning use and high temperature settings. Hence, the creation of a watchful environment has been recommended as a measure against heatstroke [29]. In Finland, mortality rates increased among low-income older people and people with dementia when temperatures exceeded 25 °C, which indicated the importance of heatstroke countermeasures [30]. For temperature control, automatic air-conditioner regulators and the use of both air conditioners and fans were recommended. However, awareness was especially important [31] and also effective in encouraging behavioral changes through recognition via a thermo-hygrometer [32]. For older adults living alone, it is especially important to promote awareness from care supporters, helpers, and care managers. Hence, education of care supporters is also necessary. Older adults become less sensitive to temperature changes, therefore it is necessary to place thermometers and hygrometers in locations where they can be easily seen and read, so that they can consciously monitor indoor temperatures. Additionally, because temperatures vary with height indoors, it is important to ensure proper air circulation by paying attention to the height of the bedding. This may require the use of fans or other devices, as well as regular temperature monitoring by care providers. Temperature and humidity control throughout the day and night, especially during the summer, is important. Therefore, the development of a system that can provide uninterrupted support throughout the day starting from the early stages of care is necessary. We hope to witness the spread of IT-based indoor temperature control systems in the future.

Although there was a significant difference in oral care, the rate of support use was not high. The utilization rate for the interrupted group was only 66.7%, even for persons at Nursing Care Level 3 or higher. In a report on oral care independence among older adults at home [33], 70% and 80% of them were independent in “brushing teeth” and “gargling”, respectively, which was consistent with the results of the continuous group in this study. A study on oral environment status [34] indicated that being at Nursing Care Level 3 or above was a factor associated with a decline in oral health. Such a decline could result in aspiration pneumonia and increase the risk of death [35]. We believe that the decline in oral health among those living alone is due to inadequate care, especially after dinner. Creating a support system to encourage tooth brushing and gargling as well as promoting regular checkups and use of visiting dentists will be necessary.

### 4.4. Limitations and Future Research

Based on the responses of the care managers, this study examined the factors that contribute to the continuation of living alone among older people who require care and live alone in urban areas. Differences in the use of LTCI and non-insurance care services and the content of IADL support were examined. When older adults who require care exhibit cognitive decline, it is difficult to obtain accurate information about their daily lives and the social resources they use, particularly when they have stopped living alone. Therefore, the survey was conducted with care managers who are responsible for planning care services, including the use of social resources. Therefore, external validity of the results was limited as it was sometimes difficult to obtain accurate information from participants regarding non-insurance care services and IADL support content. In logistic regression analysis, since the explanatory variables were limited to seven items that were highly significant, highly relevant, and likely to be confounding factors, the sample size was insufficient for the analysis. Income and educational disparities were not included in the survey items and could not be controlled for confounding factors. Since the data on the number of care-dependent individuals among urban elderly living alone are unavailable, it is impossible to determine whether the distribution of the sample in this study is representative. However, no previous studies have compared the factors that enable older adults who require care and live alone in urban areas to continue living alone, nor have any studies focused on the nursing care level required by the continuous and interrupted groups.

Future studies should increase the number of participants and expand the survey scale while also considering regional characteristics to improve the validity of the results. In addition, a further detailed study of the survey items is warranted.

## 5. Conclusions

This study aimed to clarify the effective social resources to best support the lives of older persons who require long-term care and live alone in urban areas. The use of LTCI and non-insurance care services and IADL support for older adults was examined and the issue of the nursing care level required was highlighted. Furthermore, differences in characteristics between the groups who continued to live alone and those who discontinued living alone were analyzed. The use of nursing care services and content of IADL support differed between the groups. The continuous group was characterized by a variety of support providers and frequent use of communication support such as conversation partners and assistance with outings. The interrupted group was characterized by 40% receiving medical treatment and frequent use of nursing care services. In terms of daily living support, they frequently used hygiene-related support such as temperature control, cleaning, garbage disposal, laundry, and oral care. It also became clear that the characteristics differed depending on the level of care required.

In the Support Care Level–Nursing Care Level 2 category, significant differences were observed in temperature control, oral care, medical treatment, home-visit nursing care, management guidance for in-home care, and medication management between the continuous and interrupted groups. Furthermore, in the Nursing Care Level 3–5 category, significant differences were observed in temperature control and management guidance for in-home care between continuous and interrupted groups. It was clear that the interrupted group had daily living support and used a wide variety of care services from the earliest stages of support and nursing care. Among the factors related to continuing to live alone, temperature control showed a significant difference between older adults who required light assistance and those who required heavy assistance. Logistic regression analysis also showed that temperature control influenced the continuation of living alone.

The findings of this study suggest the need to create a system that can provide uninterrupted support throughout the day, starting from the early stage when older adults start requiring care. Care workers and health professionals providing at-home care should be made aware of the importance of temperature control, especially during summer. It is necessary to place thermometers and hygrometers in appropriate locations where they can be easily seen to ensure air circulation and to promote awareness among older adults and regular temperature checks by care managers. With an aging society and shortage of care institutions, it is important to maintain older adults’ well-being and quality of life to enable them to live independently.

## Figures and Tables

**Table 1:** Characteristics of care managers.

Item		Continuous (*n* = 90) *n* (%)	Interrupted (*n* = 32) *n* (%)	*p*-Value
Age				
	40–49	35 (38.9)	16 (50.0)	0.302
	50–	55 (61.1)	16 (50.0)	
Gender				
	Female	75 (85.2)	25 (89.3)	0.758
	Male	13 (14.8)	3 (10.7)	
Years of experience				
	1–2	21 (23.3)	5 (15.6)	0.149
	3–4	16 (17.8)	9 (28.1)	
	5–9	34 (37.8)	7 (21.9)	
	10–	19 (21.1)	11 (34.4)	

Fisher’s exact test was used. The numbers in parentheses represent the ratio by denominator excluding missing values.

**Table 2:** Differences in characteristics between the continuous and interrupted groups.

Characteristics		Continuous (*n* = 90) *n* (%)	Interrupted (*n* = 32) *n* (%)	*p*-Value
Age				
	60–64	1 (1.1)	0 (0.0)	0.385
	65–69	4 (4.4)	1 (3.1)	
	70–74	7 (7.8)	1 (3.1)	
	75–79	13 (14.4)	3 (9.4)	
	80–84	19 (21.1)	5 (15.6)	
	85–89	25 (27.8)	12 (37.5)	
	90–94	15 (16.7)	4 (12.5)	
	95–99	6 (6.7)	4 (12.5)	
	100+	0 (0.0)	2 (6.2)	
Gender				
	Female	69 (79.3)	18 (64.3)	0.131
	Male	18 (20.7)	10 (35.7)	
Support/Nursing Care Level				
	Support Care Level 1	4 (4.4)	1 (3.1)	0.002
	Support Care Level 2	10 (11.1)	0 (0.0)	
	Nursing Care Level 1	21 (23.3)	3 (9.4)	
	Nursing Care Level 2	36 (40.0)	9 (28.1)	
	Nursing Care Level 3	10 (11.1)	14 (43.8)	
	Nursing Care Level 4	4 (4.4)	2 (6.2)	
	Nursing Care Level 5	5 (5.6)	3 (9.4)	
Degree of independent daily living for older adults with disabilities				
	Rank J1	6 (6.7)	0 (0.0)	0.101
	Rank J2	18 (20.2)	6 (18.8)	
	Rank A1	22 (24.7)	5 (15.6)	
	Rank A2	29 (32.6)	8 (25.0)	
	Rank B1	9 (10.1)	6 (18.8)	
	Rank B2	3 (3.4)	4 (12.5)	
	Rank C1	1 (1.1)	1 (3.1)	
	Rank C2	1 (1.1)	2 (6.2)	
Degree of independent daily living for older adults with dementia				
	Rank I and below	36 (40.4)	4 (12.5)	0.018
	Rank IIa	15 (16.9)	9 (28.1)	
	Rank IIb	23 (25.8)	10 (31.2)	
	Rank IIIa	7 (7.9)	7 (21.9)	
	Rank IIIb	3 (3.4)	1 (3.1)	
	Rank IV	4 (4.5)	0 (0.0)	
	Rank M	1 (1.1)	1 (3.1)	
Medical treatment				
	Used	16 (18.4)	12 (37.5)	0.049
	Not used	71 (81.6)	20 (62.5)	
Care supporters				
	Family members or relatives living separately	74 (83.1)	27 (84.4)	0.038
	Community residents	2 (2.2)	3 (9.4)	
	Welfare committee members	1 (1.1)	0 (0.0)	
	Care helpers	8 (9.0)	0 (0.0)	
	Friends	2 (2.2)	0 (0.0)	
	Others	2 (2.2)	0 (0.0)	
	None	0 (0.0)	2 (6.2)	

**Table 3:** Long-term care service use characteristics of the continuous and interrupted groups.

Items		Continuous (*n* = 90) *n* (%)	Interrupted (*n* = 32) *n* (%)	*p*-Value
Home-visit care				
	1/week	21 (23.3)	7 (21.9)	0.156
	2/week	21 (23.3)	4 (12.5)	
	3/week	11 (12.2)	3 (9.4)	
	4/week	3 (3.3)	4 (12.5)	
	5/week	7 (7.8)	2 (6.2)	
	6+/week	11 (12.2)	9 (28.1)	
	Not used	16 (17.8)	3 (9.4)	
Home-visit bathing service				
	6+/week	1 (1.1)	0 (0.0)	1.000
	Not used	89 (98.9)	32 (100.0)	
Home-visit nursing care				
	1/week	12 (13.3)	6 (18.8)	0.474
	2/week	6 (6.7)	4 (12.5)	
	3/week	1 (1.1)	1 (3.1)	
	5/week	1 (1.1)	0 (0.0)	
	Not used	70 (77.8)	21 (65.6)	
Management guidance for in-home care				
	2/month	5 (5.6)	1 (3.1)	<0.001
	4+/month	1 (1.1)	9 (28.1)	
	Not used	84 (93.3)	22 (68.8)	
Commuting for care				
	1/week	6 (6.7)	1 (3.1)	0.071
	2/week	19 (21.1)	7 (21.9)	
	3/week	10 (11.1)	2 (6.2)	
	4/week	0 (0.0)	2 (6.2)	
	5/week	4 (4.4)	0 (0.0)	
	6/week	1 (1.1)	3 (9.4)	
	Not used	50 (55.6)	17 (53.1)	
Commuting for rehabilitation services				
	1/week	5 (5.6)	0 (0.0)	0.189
	2/week	10 (11.1)	3 (9.4)	
	3/week	2 (2.2)	4 (12.5)	
	4/week	1 (1.1)	0 (0.0)	
	5/week	1 (1.1)	0 (0.0)	
	Not used	71 (78.9)	25 (78.1)	
Home-visit rehabilitation				
	1/week	9 (10.0)	0 (0.0)	0.068
	2/week	1 (1.1)	2 (6.2)	
	3/week	1 (1.1)	0 (0.0)	
	Not used	79 (87.8)	30 (93.8)	
Short-term stay at a care facility				
	3+/month	1 (1.1)	0 (0.0)	0.097
	1–2/month	4 (4.4)	5 (15.6)	
	6/Year	1 (1.1)	1 (3.1)	
	Not used	84 (93.3)	26 (81.2)	
Temporary provision of assistive products				
	Not used	34 (37.8)	5 (15.6)	0.027
	Used	56 (62.2)	27 (84.4)	
House modification				
	Not used	67 (75.3)	18 (62.1)	0.233
	Used	22 (24.7)	11 (37.9)	
Meal delivery service				
	Not used	73 (81.1)	21 (65.6)	0.045
	1/week	0 (0.0)	0 (0.0)	
	2–3/week	8 (8.9)	1 (3.1)	
	4–6/week	5 (5.6)	6 (18.8)	
	1/day	3 (3.3)	3 (9.4)	
	2+/day	1 (1.1)	1 (3.1)	
Eating out				
	Not used	73 (81.1)	29 (90.6)	0.804
	1/week	7 (7.8)	2 (6.2)	
	2–3/week	5 (5.6)	0 (0.0)	
	4–6/week	1 (1.1)	0 (0.0)	
	1/day	4 (4.4)	1 (3.1)	
Use of take-out lunch boxes and prepared foods				
	Not used	48 (53.3)	15 (46.9)	0.691
	1/week	2 (2.2)	1 (3.1)	
	2–3/week	26 (28.9)	10 (31.2)	
	4–6/week	8 (8.9)	5 (15.6)	
	1/day	4 (4.4)	0 (0.0)	
	2+/day	2 (2.2)	1 (3.1)	
Emergency alert equipment				
	Not used	86 (98.9)	26 (83.9)	0.005
	Used	1 (1.1)	5 (16.1)	
Nursing care prevention support				
	Not used	81 (90.0)	29 (90.6)	0.379
	Exercises	1 (1.1)	1 (3.1)	
	Fun activities	3 (3.3)	0 (0.0)	
	Social event	3 (3.3)	0 (0.0)	
	Others	2 (2.2)	2 (6.2)	

Fisher’s exact test was used. The numbers in parentheses represent the ratio by denominator excluding missing values.

**Table 4:** Differences in support between the continuous and interrupted groups regarding IADL.

Details of Support	Continuous (*n* = 90) *n* (%)	Interrupted (*n* = 32) *n* (%)	*p*-Value
Talking to others	82 (91.1)	30 (96.8)	0.445
Frequency of outings	81 (90.0)	24 (77.4)	0.120
Cleaning	75 (85.2)	28 (90.3)	0.558
Garbage disposal	52 (58.4)	25 (80.6)	0.030
Medication management	45 (50.6)	24 (77.4)	0.011
Money management	42 (46.7)	21 (67.7)	0.060
Laundry	39 (44.3)	21 (67.7)	0.036
Meal preparation	37 (41.6)	22 (71.0)	0.006
Temperature control	26 (28.9)	27 (87.1)	<0.001
Sanitary	22 (24.7)	12 (38.7)	0.166
Oral care	16 (17.8)	18 (58.1)	<0.001

Fisher’s exact test was used. The numbers in parentheses represent the ratio by denominator excluding missing values.

**Table 5:** Differences between the Support Care Level 1–Nursing Care Level 2 and Nursing Care Level 3–5 categories.

Item		Support Care Level 1–2 and Nursing Care Level 1–2 (*n* = 84)			Nursing Care Level 3–5 (*n* = 38)		
		Continuous (*n* = 71) *n* (%)	Interrupted (*n* = 13) *n* (%)	*p*-Value	Continuous (*n* = 19) *n* (%)	Interrupted (*n* = 19) *n* (%)	*p*-Value
Characteristics							
Medical treatment							
	Used	10 (14.3)	6 (46.2)	0.016	6 (35.3)	6 (31.6)	1.000
	Not used	60 (85.7)	7 (53.8)		11 (64.7)	13 (68.4)	
Care services							
Management guidance for in-home care							
	2/month	3 (4.2)	0 (0.0)	0.019	2 (10.5)	1 (5.3)	0.020
	4/month	1 (1.4)	3 (23.1)		0 (0.0)	6 (31.6)	
	Not used	67 (94.4)	10 (76.9)		17 (89.5)	12 (63.2)	
Home-visit nursing care							
	1/week	8 (11.3)	5 (38.5)	0.022	4 (21.1)	1 (5.3)	0.626
	2/week	4 (5.6)	2 (15.4)		2 (10.5)	2 (10.5)	
	5/week	1 (1.4)	0 (0.0)		1 (5.3)	1 (5.3)	
	Not used	58 (81.7)	6 (46.2)		12 (63.2)	15 (78.9)	
IADL support							
Temperature control							
		17 (23.9)	10 (76.9)	<0.001	9 (47.4)	17 (94.4)	0.003
Oral care							
		8 (11.3)	6 (46.2)	0.007	8 (42.1)	12 (66.7)	0.191
Medication management							
		30 (42.9)	10 (76.9)	0.034	15 (78.9)	14 (77.8)	1.000

Fisher’s exact test was used. The numbers in parentheses represent the ratio by denominator excluding missing values.

**Table 6:** Factors affecting the continuation of living alone among older people.

Variable	Odds Ratios	95%CI	*p*-Value	VIF
(Intercept)	693.00	9.79–49,000.00	0.003	
Support/Nursing Care Level	0.80	0.52–1.23	0.299	1.298
Degree of independent daily living for older adults with dementia	1.53	0.95–2.48	0.084	1.616
Management guidance for in-home care	1.32	0.92–1.87	0.129	1.038
Emergency alert equipment	0.14	0.01–1.76	0.129	1.114
Temperature control	0.06	0.01–0.24	<0.001	1.358
Oral care	0.52	0.15–1.79	0.300	1.424
Meal preparation	1.08	0.32–3.70	0.901	1.363

Likelihood ratio test: *p* < 0.001.

## Data Availability

Restrictions apply to the availability of these data due to privacy and ethical reasons.
